# Supplementary material for: Lacticaseibacillus casei Strain Shirota Modulates Macrophage-Intestinal Epithelial Cell Co-Culture Barrier Integrity, Bacterial Sensing and Inflammatory Cytokines
Source: Microorganisms. 2022 Oct 21;10(10):2087. doi: 10.3390/microorganisms10102087 (PMC9607601; doi:10.3390/microorganisms10102087)
Supplement: Supplementary file 1 [file microorganisms-10-02087-s001.zip › microorganisms-1904785-supplementary.pdf]

Supplementary Section:

Supplementary Table S1. Supporting evidence for PMA- and VD3-differentiated THP-1 derived M1/M2 macrophage subset cell model corresponding to IFN-γ/LPS and IL-4/IL-13-differentiated THP-1 and primary cell studies.

**Supporting evidence for THP-1 cell model**		
	PMA/M1 Subset	VD3/M2 Subset
Gene Expression	Arginase$^{neg/lo}$<br>CD206$^{neg/lo}$<br><br>IL-8^{hi}<br>IL-10^{lo}<br>IL-12p40^{hi}<br>iNOS^{hi}<br>TNF-α^{hi}	Arginase^{hi}<br>CD206^{hi}<br>DC-SIGN^{hi}<br>IL-8^{lo}<br>IL-10^{hi}<br><br>iNOS$^{neg/lo}$<br>TNF-α^{lo}
Functional Responses to LPS stimulation	IL-10^{lo}<br>IL-12^{hi}<br>Phagocytosis^{lo}<br>Superoxide^{hi}<br>TNF-α^{hi}<br>IL-1β^{hi}<br>IL-6^{hi}<br>IL-8^{hi}	IL-10$^{hi/med}$<br>IL-12$^{neg/lo}$<br>Phagocytosis^{hi}<br>Superoxide^{lo}<br>TNF-α^{lo}<br>IL-1β^{lo}<br>IL-6^{lo}<br>IL-8^{lo}
Surface Markers	CCR1^{+}<br>CCR2^{lo}<br>CD206^{lo}<br>TLR4^{hi}	 <br>CD206^{hi}
Proteomic Profiling	CCL20 isoform 2<br>ICAM-1<br>IL-1β<br>SOD isoform 4<br>TLR2<br>TNF-α induced protein 8	CD68<br>C-type mannose receptor 2<br>TGF-β_1<br>Integrin αM<br>Macrophage capping protein,<br>Myeloid cell nuclear differentiation antigen
Reproduced Observations	THP-1 + IFNγ/LPS treatment	THP-1 + IL-4 treatment
